# Neurofeedback and the Neural Representation of Self: Lessons From Awake State and Sleep

**DOI:** 10.3389/fnhum.2018.00142

**Published:** 2018-04-13

**Authors:** Andreas A. Ioannides

**Affiliations:** Laboratory for Human Brain Dynamics, AAI Scientific Cultural Services Ltd., Nicosia, Cyprus

**Keywords:** default mode network, zone of proximal development (ZPD), electroencephalography (EEG), magnetoencephalography (MEG), resting state networks (RSN), nature of dreams, neural representation of self, mothering effect

## Abstract

Neurofeedback has been around for half a century, but despite some promising results it is not yet widely appreciated. Recently, some of the concerns about neurofeedback have been addressed with functional magnetic resonance imaging and magnetoencephalography adding their contributions to the long history of neurofeedback with electroencephalography. Attempts to address other concerns related to methodological issues with new experiments and meta-analysis of earlier studies, have opened up new questions about its efficacy. A key concern about neurofeedback is the missing framework to explain how improvements in very different and apparently unrelated conditions are achieved. Recent advances in neuroscience begin to address this concern. A particularly promising approach is the analysis of resting state of fMRI data, which has revealed robust covariations in brain networks that maintain their integrity in sleep and even anesthesia. Aberrant activity in three brain wide networks (i.e., the default mode, central executive and salience networks) has been associated with a number of psychiatric disorders. Recent publications have also suggested that neurofeedback guides the restoration of “normal” activity in these three networks. Using very recent results from our analysis of whole night MEG sleep data together with key concepts from developmental psychology, cloaked in modern neuroscience terms, a theoretical framework is proposed for a neural representation of the self, located at the core of a double onion-like structure of the default mode network. This framework fits a number of old and recent neuroscientific findings, and unites the way attention and memory operate in awake state and during sleep. In the process, safeguards are uncovered, put in place by evolution, before any interference with the core representation of self can proceed. Within this framework, neurofeedback is seen as set of methods for restoration of aberrant activity in large scale networks. The framework also admits quantitative measures of improvements to be made by personalized neurofeedback protocols. Finally, viewed through the framework developed, neurofeedback’s safe nature is revealed while raising some concerns for interventions that attempt to alter the neural self-representation bypassing the safeguards evolution has put in place.

## Introduction

Each one of us likes to be in control. Illness, and specifically psychological disturbances, compromises our mental resources to exercise control. In this weakened state we are at the hands of medical experts trained to treat symptoms with pharmaceuticals that reduce or abolish them. Severe, and sometimes not so severe, psychological problems are addressed with medication affecting the basic biochemistry of the brain. Over time, such medication may lead to re-adjustments of the brain biochemistry and/or introduce unwanted side effects (Ketter et al., 1999; Correl and Carlson, 2006). In such situations, any method that can provide effective solutions, by recruiting the (putative) remarkable ability of the brain to self-regulate its activity in a goal directed way, has an intrinsic appeal with patients and carers alike. The perception is strong enough in the minds of patients to affect treatment selection and effectiveness (van Schaik et al., 2004). Control of physiological processes is often implicit and it can occur at the level of individuals or groups, thus playing a facilitating role in emotional understanding and group coherence (Cooper et al., 2014). Biofeedback is a broad family of methods that claim to do just that, through greater awareness and eventually control of physiological processes like brain activity (Kober et al., 2013), muscle tone (Giggins et al., 2013), skin conductance (Kotwas et al., 2017), heart rate (Lehrer and Gevirtz, 2014), and pain perception (Jensen et al., 2014). In this paper the focus is placed on the control over brain activity, namely neurofeedback (NF). In a nutshell, NF is an umbrella term for methods using recordings of ongoing brain activity as a tool for self-regulation of brain function (Sitaram et al., 2016). For many years, excitement and skepticism about NF co-existed within separate communities that rarely interacted with each other. In the next two sections, the current state of neurofeedback is reviewed. Impressive looking claims by NF were ignored as largely anecdotal reports that did not contain the usual prerequisites of control, double blind and carefully controlled procedures expected in traditional science and clinical studies.

In this paper, some of recent studies that have provided new insights, but have not yet clarified the scene, are first briefly reviewed. It is nevertheless noted that fully resolving all uncertainties is not the primary goal of this paper. Before attempting to resolve apparently conflicting outcomes, it is important to establish a framework for asking the right questions. Satisfying this prerequisite is the overall goal of this work and it will be approached through the following three objectives. First, to provide some important background on NF and clarify why there is a fundamental difference in approach between NF and traditional neuroscience (see section “Difference in Approach Between Traditional (Neuro)science and Neurofeedback”). These differences in intrinsic approaches are then set against the results of recent studies. Some of these results support NF while others fail to find evidence for NF efficacy (see section “Evidence for and Against Neurofeedback Efficacy”). The second objective of the paper is to allow the apparently irreconcilable approaches of NF and traditional (neuro)science and medicine to be examined under a common and scientifically acceptable theoretical framework. This theoretical framework is assembled with key concepts from psychology that were originally used for describing the development of a human child; these concepts are generalized and cloaked with current neuroscientific knowledge, a process that brings to the forefront a key neural system that has the hallmarks of the core neural representation of self (see section “A Theoretical Framework”). In section “The Living Self Within and Beyond the Zone of Proximal Development” cases are reviewed when this neural representation of self is supported by existing mechanisms (as described in section “A Theoretical Framework”) and when it is not. In the final section “What Neurofeedback Is Like (The Mothering Effect),” the theoretical framework is used to relate NF with other well understood human activities and hence provide a way for bridging the conceptual gap that so far separates NF approaches from those of traditional (neuro)science and medicine.

## Difference in Approach Between Traditional (Neuro)Science and Neurofeedback

The common thread of the many NF methods is the use of actual measurements of brain activity in real time to promote self-regulation of brain function. The measurements, or some features derived from them, are presented to the subject whose task is to modify the brain activity in a specific direction. This modification can be achieved with conscious effort by the subject. Alternatively, the measurements are used to change the auditory or visual stream as it is presented to the subject: features corresponding to increases in “good” brain activity are rewarded through pleasant changes in the visual and/or audio streams. Features corresponding to increases in “bad” brain activity are punished through disagreeable changes in the visual and/or audio streams. The brain picks on these contingencies, even when the subject is not aware of either every change in the visual or auditory stream or the contingencies of the changes to the variations in his/her brain activity. For many years, electroencephalography (EEG) provided the main measurement on which NF was based (Angelakis et al., 2007). Although EEG provides a direct correlate of mass electrical activity in the brain, the use of EEG signal as the basis for providing NF encountered stiff resistance because it was not easy to relate it to mechanisms that could explain the reported results. To understand the reasons for this skepticism, we highlight the difference between traditional neuroscience experiments and typical NF approach. The comparison is a little stretched because a NF session is not (should not be) an experiment, but a consultation. It is posed in this way, however, because this is how it is often treated when the results are to be described along the results of traditional science.

In a typical neuroscience experiment a precise protocol is used to find what is common in the population across individuals or, differs across distinct groups of people. The individual subject is insignificant in the overall scheme of things. Any one subject is treated in the same way as any other through precise instructions about how to deal with the stimuli and what strategy to follow. There is some control of the general state of the subject and his/her preoccupations at the time of the experiment, but the subject is asked to behave in a stereotypical way and he/she is not encouraged to become engaged with the overall experience. During the active part of the experiment, each subject is instructed what to do, which stimuli to pay attention to and which to ignore. In many experiments, the subject is also instructed to “empty” the mind and to “do nothing” for the duration of any baseline conditions that will be used as reference. The justification for using such experiments to probe what our brains actually do in the real world is weak for a number of reasons. To begin with, evolution has shaped us to be always engaged with our environment while awake, looking for novelty and change and to ignore the regular and expected. The natural tendency is to treat stimuli that are repeated in identical way many times, as aspects of the environment to be ignored. The actual response elicited by typical stimuli in an experiment is minute and it is interwoven in a non-linear way to the much stronger background generated by ongoing brain processes. When we have “nothing to do” our brain is not idle, but it is actually busy putting in some order recent events, planning what to do next, or anticipating happy or not so happy future encounters. Therefore, it is more likely that the evoked response, one so carefully measures, is determined, at least partially, by brain processes attempting to override the natural tendencies that the stimuli would normally elicit.

During a NF session the individual subject is the center of everything that goes on. His/her mental and emotional state, wishes, motivation and the reasons for attending the NF session (should) determine what will take place. The reward/punishment signal can be presented in any one of different ways: it could be a bar, a color on the screen or changes in how a racing car runs the course or the level and properties of background sounds and it is/should be adjusted for each individual for maximum effect. What is critical is that the outcome is achieved, either through a conscious control of the stimulus or through a subconscious learning of the contingencies between the targeted good and bad features of brain activity and the changes in the visual or auditory streams.

Therefore there is a stark contrast between doing a “proper” neuroscience experiment and an “effective” NF session. In the former case, everything must be controlled and any deviation from the fixed protocol makes the experiment invalid. In NF, the conditions from one session to the other, even the instructions should be modified to maximize the desired effect. Information (should be) accumulated, during the NF session and in the days between NF sessions about how the subject responded to the last and previous NF interventions. The evaluation of this information should guide decisions about how the next session is to be conducted. If the evaluation suggests that a change in the protocol or in the way the subject is instructed is likely to bring the desired effect faster, then this change should be immediately implemented; it makes no sense, not to make these changes for the sake of sticking to a fixed protocol. This brings again the unfairness of the comparison, so we will end this section by considering a more appropriate example, one that is more similar to a clinical case than a neuroscience experiment. A child with some attention or cognitive problem is in the middle of a NF intervention scheduled for 10 NF sessions. The protocol in each NF session provides reward and punishment through changes in the visual and auditory content in two scenarios, each of equal duration. The theme for the first scenario is from a train ride and the second from a car race. Suppose that half way, i.e., after the fifth session, the child has improved a little, in some measurable and quantifiable way. It nevertheless becomes noticeable in session 5 that the child is losing interest and is almost asleep during the train ride scenario. How should the experiment proceed in session 6? Should only the car race scenario be used, or possibly another scenario introduced, if available, to replace the one with the train ride? Alternatively, should the same scenario continue because if it is changed, then the results cannot be included in some grand analysis that will lead to a publication? To this ethical dilemma, NF practitioners have opted for the choice that maximizes the benefit to the child. The challenge for the field is how best to advance our understanding of the underlying processes that take place during a routine NF sessions (where protocols can change from one session to the next) and from NF experiments where the same fixed protocol is applied on subject cohorts.

## Evidence for and Against Neurofeedback Efficacy

The first detailed work on what we now call NF started in the late 1950s and early 1960s. All the early work on NF, and almost all work that followed until the last decade of the twentieth century, used electroencephalography (EEG) focusing on controlling one’s own EEG rhythms. Early NF research produced some remarkable results (Kamiya, 1968; Sterman and Friar, 1972). However, these results did not influence the wider scientific community and some of them only recently became widely accessible (Sterman et al., 2010; Kamiya, 2011). The NF approach simply did not fit the accepted orthodoxy about how the brain works and how its problems are to be treated. Rhythms of brain electrical activity were on the fringe, except for the researchers and clinicians working on sleep and epilepsy, two areas that had a great impact on the early work of neurofeedback (Kamiya, 2011). This is not surprising, because sleep and epilepsy are the two well-known conditions where the collective electrical activity gives rise to large enough EEG features. The resulting large EEG graphoelements were just about the only features that could be recognized with the EEG instrumentation available at the time. The drive at the time was to employ techniques like averaging to separate out what was believed to be the true brain signal from the nebulous concept of “brain noise.” The prevailing view was that rhythms of the brain were part of the brain noise and that one could not control the rhythms generated by the mass electrical activity in one’s brain either for its own sake or as a way of affecting physiology and/or behavior. The error of this belief was not recognized for a long time, despite the fact that, as long as nearly fifty years ago the ability to control the activity of a single neuron was demonstrated in animals (Fetz, 1969) and only much latter in humans (Cerf et al., 2010). The impasse was upset in the last decade of the twentieth century for a combination of reasons that will be describe next.

### Support for NF: Direct Modulation/Access of Brain Areas and Network-Wide Oscillations

Recent support for NF has come from four quarters. First, real time fMRI (rtfMRI) demonstrated that it is indeed possible to train subjects to change the level of activity of well-defined brain areas, for example rostral–ventral and dorsal part of the anterior cingulate cortex (ACC) (Weiskopf et al., 2003). More importantly, it was also demonstrated that training patients with various afflictions to change activity in specific brain areas (where one finds aberrant activity for their condition) had beneficial effects, for example, training depressed patients to down-regulate responses from the saliency network, mainly the fronto-insular cortex and dorsal ACC (Hamilton et al., 2016). For attention deficit hyperactivity disorder (ADHD) improvements were seen after successful training to up-regulate the dorsal ACC (dACC) for adults (Zilverstand et al., 2017), and the right inferior prefrontal cortex for adolescents (Alegria et al., 2017). Improvements in cognitive function (Zilverstand et al., 2017) and significant reduction in ADHD symptoms were documented both soon after neurotherapy and at 11-month follow-up (Alegria et al., 2017). Parkinson patients were trained to increase the activity in their supplementary motor area (SMA) that lead to successful increases in SMA itself and other areas connected to the SMA with concomitant improvements in motor speed and clinical ratings of motor symptoms (Subramanian et al., 2011). Over the last few years, the number of studies with real time fMRI (rtfMRI), the areas targeted and the clinical conditions increase rapidly with time. With online-feedback using decoded fMRI signals, it is possible to improve visual perceptual learning (Shibata et al., 2011) and reduce fear conditioning (Koizumi et al., 2016), while the subjects remain unaware of the content and purpose of the procedure.

Second, magnetoencephalography (MEG) has entered the neurofeedback field while localization of generators with EEG has allowed the incorporation of localization algorithms and especially sLORETA (Pascual-Marqui, 2002) instead of the well-established few electrode or frequency dependent EEG neurofeedback. With MEG it is possible to target superficial brain areas with judicial selection of linear combinations of MEG channels, or just a single channel for the planar gradiometer coil design. These developments have allowed researchers to apply area-targeted neurofeedback using MEG (Okazaki et al., 2015) and EEG (Liechti et al., 2012; Bauer and Pllana, 2014).

Third, it is now abundantly clear that inducing changes in electrical activity in targeted brain areas or large cortical regions can lead to lasting changes in brain activity (at the targeted areas and more widely), change aspects of behavior and mood and reverse pathology. The targeted changes in brain activity can be made by applying direct or alternating electrical current on the scalp (Antal and Paulus, 2012), or by inducing currents in the brain by applying single, multiple, or repetitive magnetic pulses from just outside the brain (Wassermann and Lisanby, 2001; Lee et al., 2012). Using devices on the scalp or just outside has the advantage that it is minimally invasive, but at the expense of low spatial specificity about exactly what area is affected and the effect in surrounding areas. Nevertheless with a range of such techniques it is possible to induce changes in the brain with potentially clinical improvements in a variety of conditions.

Finally, fully invasive procedures and especially deep brain stimulation (DBS) demonstrated that with precise targeting of deep brain nuclei and refined frequency control for the stimulation dramatic and seemingly irreversible loss of function can be restored. Today, DBS has transformed the treatment of a number of disorders for patients with otherwise treatment-resistant movement and affective disorders (Kringelbach et al., 2007).

These diverse sets of results demonstrated that control of local brain activity or oscillations can modulate large brain networks, with a cascade of linked interactions over a very broad frequency range and spatial dimensions. The role of oscillations and more generally temporal correlations in linking activity in the brain and cognition has a long history. The most recent history can be traced to the demonstration of autorhythmic electrical oscillatory properties of individual neurons (Llinas, 1988). In recent years the field increasingly appreciates the importance of temporal correlations of neural activity within and across cortical areas (Singer and Gray, 1995; Castelo-Branco et al., 1998) and deep brain centers (Courtemanche et al., 2013). It is recognized that large scale networks mediated by coherence-based dynamic links (Varela et al., 2001) and consolidated by synaptic plasticity are an integral part of mechanisms supporting temporal representation and long-term consolidation of information (Buzsáki and Draguhn, 2006). With this accumulated evidence the foundation for NF to influence brain activity is plausible, although there is still much work to be done to identify the mechanisms involved and understand their details.

### Mixed Evidence for NF Efficacy

Recent years has seen new EEG NF studies allowing quantitative evaluation of outcomes and in some cases employing double blind, placebo control designs. These studies are expensive and therefore are usually undertaken by researchers in academic institutions, as part of funded research. Gruzelier and colleagues set the standards with a series of studies providing quantitative evidence of NF-induced improvements in the creative performance of musicians and artists, for a review see (Gruzelier, 2014a,b,c). In the last few years, these pioneering studies were followed by considerable volume of work that included meta-analysis of earlier studies and new studies that had the controls expected in traditional scientific investigations, but a firm conclusion about the efficacy of NF has not yet been reached. In the rest of this subsection two areas targeted by recent NF studies will be examined, Attention-Deficit/Hyperactivity Disorder (ADHD) and insomnia.

Arguably NF intervention is most often applied to ADHD cases. This is not surprising since a good classification of ADHD is possible using EEG features (Monastra et al., 1999). There are a range of NF protocols promoted for ADHD and this poses a real challenge when one tries to evaluate the efficacy of the intervention in general. Early results (Lubar et al., 1995) and a number of recent meta-analysis (Arns et al., 2014; Hodgson et al., 2014) provided support for claims of NF efficacy both in terms of improvements in symptoms and school performance in children with ADHD. Very recently though, careful studies, using double-blind, sham-controlled designs for EEG NF of ADHD (Arnold et al., 2013; Vollebregt et al., 2014) and meta-analysis of controlled NF studies. (Vollebregt et al., 2014; Cortese et al., 2016) produced less favorable results. The two meta-analysis studies reach the same negative conclusion, failing to either find “any benefit of neurofeedback on neurocognitive functioning in ADHD” (Vollebregt et al., 2014) or “to support neurofeedback as an effective treatment for ADHD” (Cortese et al., 2016). The reasons for the rather negative outcome are likely to be a combination of factors, including methodological issues (at the level of NF intervention and the methods used for the evaluation of NF results from different interventions), the diversity of approaches used for ADHD EEG NF (Zuberer et al., 2015) and the different sub-types of ADHD (Vernon et al., 2008). Below I will discuss the case of NF approaches to insomnia, where although a similar negative outcome is encountered, the more limited range of NF methods make it easier to examine the reasons for the apparent inconsistencies.

The rationale for EEG NF as an intervention for insomnia goes back to the very beginning of NF. Increasing the EEG in the 11–15 Hz range, known as sigma band or sensorimotor rhythm (SMR) has been one of the pioneering ideas heralding the beginning of the NF era. During the awake state, EEG activity in the sigma band is recorded above and around the central sulcus before or in anticipation of movement. Activity in the sigma band is also one of the hallmarks of light sleep, seen as highly rhythmic activity, called spindles, in the EEG; spindles in the lower part of the sigma range are seen more in frontal electrodes, while spindles in the higher end of the range are seen in the same areas as SMR and in posterior parietal areas. NF training enhancing SMR has been directly related to increased relaxation and improvements in sleep, including increase in spindle activity (Sterman et al., 1970). There were, however, relatively few studies of possible applications to insomnia in the early days, with most of these using primarily biofeedback (Hauri, 1981; Hauri et al., 1982; Morin et al., 1999). More recently Hoedlmoser et al. (2008, 2014), Cortoos et al. (2010), and Schabus et al. (2017) performed new NF experiments focusing on SMR training. In their first studies, Hoedlmoser et al. (2008) reported positive effects of NF SMR training in young healthy individuals and young patients with subclinical insomnia (Hoedlmoser et al., 2014). The results indicated a beneficial effect after only 10 sessions of 12–15 Hz SMR NF training. Improvements were found for sleep quality and memory performance (Hoedlmoser et al., 2008; Schabus et al., 2014) and for overnight memory consolidation. Cortoos has added two innovations to earlier interventions of insomnia, using a design that employed both biofeedback and NF (Cortoos et al., 2010). First, subjects were trained at home with a secure internet connection. Second, a novel NF protocol was introduced, reinforcing sensorimotor rhythm (SMR) and inhibiting theta and high beta power. In addition to the NF group, a biofeedback group was included with the task to decrease electromyographic activity at the Fpz electrode (power in the 5–70 Hz range) that was assumed to be equivalent to reinforcement of relaxation. The subjects slept for two nights in the laboratory for polysomnography, one before and one after a 20 session of either NF (*n* = 9) or Biofeedback (*n* = 8) at home, spread over a period of 8 weeks. Comparison of objective sleep parameters from polysomnographic data demonstrated improvement in sleep onset latency for both the NF and biofeedback groups, but improvement in total sleep time and increase in REM sleep only for the NF group. Analysis of sleep diaries kept by the subjects at home, revealed an improvement on all sleep variables only for the NF group. The sleep diaries at the laboratory did not reveal any change between the first (baseline) and last (after NF and biofeedback sessions) sleep nights.

The latest study (Schabus et al., 2017) was the most refined and rigorous study of NF intervention yet, using a sample of patients with primary insomnia. This study used a counterbalanced double-blind cross-over, placebo control design with a NF task (NFT) and a placebo feedback task (PFT). A number of further additions were introduced to improve earlier designs, clarify open questions. The results of this study are mixed: they report that SMR NF training positively affects subjective measures, yet is ineffective in changing objective parameters such as spectral EEG measures, sleep architecture or memory performance. More specifically, the Schabus study found that participants can indeed obtain control of otherwise unconscious neural processes (in the 12–15 Hz or SMR oscillations). They also reported that young healthy subjects in the neurofeedback control group appear to learn more quickly and exhibit steeper learning curves than insomnia patients. The authors interpreted as a negative finding for NF specificity, the fact that in their study the learning effect was not limited to the SMR rhythm but it was also evident in the sham or PFT condition (with 15–20 Hz Beta enhancements). The key negative finding of the study is that the subjective changes reported in sleep quality were not accompanied by objective EEG-derived measures of sleep quality, contrary to expectations based on the earlier studies by the authors (Hoedlmoser et al., 2008; Schabus et al., 2014) and others (Cortoos et al., 2010). The authors suggest as explanation the more severe nature of the insomnia symptoms and the higher age in the current study and/or the inherent risk that that laboratory staff and experimenters may inadvertently bring about the desired effects in single-blind designs. These are indeed plausible explanations. One can also point to differences in the protocol between the studies as equally plausible explanations for the discrepancies. One example of a difference in the protocols is the active role played by the introduction of the PFT in the protocol. Both the NFT and PFT lead to learning in awake state. Normal controls learn faster than the subjects with insomnia complain. It is likely that more sessions are needed to increase SMR in a different state (light sleep) than the state at the time of training. It is also likely that the inclusion of an irrelevant component (PFT) interfered with the transition of the learning to changes during sleep, a view supported by the fact that PFT was also learnt. Another example of a difference is the instruction to subjects about how to achieve the increase in SMR. In the earlier study (Hoedlmoser et al., 2008), subjects were encouraged to look themselves for appropriate strategies like physiological relaxation combined with positive mental activity, while in the latest study trials with artifacts were abandoned and a new trial was started automatically. If the successful (neural activity) corresponding to successful increase in SMR was accompanied by eye movement, or other movement, it is conceivable that stopping the trial could correspond to inhibiting such learning to take place. Another important difference in the two protocols, is the component of inhibiting frequency bands (i.e., well outside the reward frequency band) in the Cortoos study (Cortoos et al., 2010) and the absence of such component, with the additional confounding effect of rewarding other frequencies in different trials (the PFT part of the protocol) in the latest Schabus study (Schabus et al., 2017).

### A Way Forward From This Conundrum

The take home message from the discussion of the insomnia studies in the last sub-section is that when one considers the evidence, one must always remember that neither success nor failure of one type of NF intervention can be generalized as evidence applicable to all the other NF interventions, without making sure that the consequences from each possible change in the protocols have been properly accounted for. Clearly to do that for ADHD and the many other putative applications of NF is a herculean task; it is nevertheless a task that the field must undertake. One of the difficulties in moving on with the task is the absence of a reliable theoretical framework for discussing NF amongst the many disciplines involved, and specifically neuroscience, psychiatry and psychology. The remainder of this paper is devoted to the development of such a framework and linking it to well known (not necessarily well understood) activities that may serve as exemplars for what NF actually does.

## A Theoretical Framework

Developing the new theoretical framework starts with the key concepts of assimilation and accommodation as originally used by Piaget and Vygotsky’s zone of proximal development (ZPD). Piaget and Vygotsky and their followers used these terms as general tools for describing psychological mechanisms in the context of learning in children; the discussion often focused on the way play influences learning which in turn shapes the individual child’s identity and its relation to its social group (Nicolopoulou, 1993). These same terms have a wider utility and they are applicable to any situation where a biological organism deals with a changing environment. As we will see in this section, the full explanatory power of these concepts becomes apparent when they are cloaked with precise neuroscience knowledge. For the purposes of this paper, these terms become tools for understanding what is happening routinely in children and adults of all ages, as they interact with their physical and social environment and as they learn from each experience. Results from recent studies are added to transform assimilation and accommodation into conduits for adaptation and learning and explore how they help us understand under a unified framework different processes and specifically attention, memory and sleep. As we will see in the remainder of this and the next section, these processes become the pillars for adaptation and learning and their *modus operandi* consistent with a prime directive of preserving as much as possible the core self-identity.

### An Interim Definition for Assimilation and Accommodation

We begin the construction of the new framework with two interim definitions that will serve for the purposes of this work as generalizations for assimilation and accommodation. Assimilation is defined as any interaction with the environment that corresponds to a (partial) fit of the experience into the current internal model of the world as this is represented by the activity of the neural networks of an individual’s brain. Such an interaction might be for example a physical action where a learnt motor sequence is recruited (often subconsciously and automatically) to deal with the new situation. Alternatively, the interaction may have an entirely cognitive nature where reading about or observing a phenomenon leads to a better understanding of its nature and its relation to other phenomena, but does not relate to revisions of our self-image; such events can be incorporated into our memory effortlessly and without affecting our emotional state. Assimilation events that cannot be fitted fully into existing internal models of the world and/or have a direct impact on our own role in the world (e.g., they are life threatening or rewarding) are emotionally labeled in the neural machinery for later more detailed processing.

Accommodation is defined as any process that requires some modification of the internal model of the world to “accommodate” new experiences that could not be (fully) accounted for during the first encounter. Under the framework we propose, accommodation may involve some major change in the internal representation of the world but only little or no re-adjustment of the part that influences or contains our own self-image. Although elements of assimilation and accommodation are present all the time, there is a clear change in emphasis in the extremes of active waking activity and sleep: assimilation dominates the awake state while accommodation is emphasized during rest and especially sleep.

### Attention and Memory in Awake State and Sleep

Assimilation and accommodation rely on attention and memory. During assimilation, attentional mechanisms filter the inputs from the environment and multiple memory mechanisms recover related stored information for comparison and action preparation. The details of the events encountered during assimilation are dissipated except for ones that are considered salient and/or novel enough that are maintained for some time in the hippocampus and amygdala and surrounding cortex (Thompson and Kim, 1996; Phelps, 2004). During accommodation, attentional mechanisms balance risks and eventually shut down any input from the environment; then and only then the transfer and consolidation of memories stored in the depths of the temporal lobe can be retrieved and transferred, via the medial prefrontal cortex (Preston et al., 2013), over the wider expanse of the cortex. Our recent results (Ioannides et al., 2009, 2017) highlight the role of the posterior parietal cortex in the process, an area known to be a key player in memory consolidation and retrieval (Izquierdo et al., 1997; Ciaramelli et al., 2008) and to act as a short term storage of limited capacity for an online but impoverished representation of visual scenes (Todd and Marois, 2004), with poor understanding though of the precise details of the underlying mechanisms and its overall role. At all times during the interaction with the environment and during rest and sleep, attention and memory act together to ensure that assimilation and accommodation are effectively implemented. During the day, awake assimilation is dominant with only the gathering of information and placement of salient events to a temporal storage relevant for future use for accommodation. During the evening, assimilation maintains priority when the environment is not completely safe, but once safety is assured, or at least can be assumed, assimilation is diminished but not fully abolished as sleep takes over. As we will see, the succession of well-defined sleep stages and transitions between them involve very specific operations related to assimilation and accommodation.

### Attention and Memory During Awake State

While awake, assimilation has precedence because the priority is to navigate safely through the environment. The internal model of the world must be continuously compared with the external reality and, if necessary, updated by the input from the senses to correctly evaluate threats and opportunities on-line as they are encountered. The flood of fragmented information from the senses is simply too much to be processed effectively, so the input must be prioritized on-line either for immediate action (avoid an obstacle while running) or put in temporal storage for a later, more detailed analysis. In any case most of the input from the senses is largely repetitive and represents neither threat nor opportunity, so evolution has ensured that its influence simply dissipates away. The process is a continuous drive for an optimal selection and integration of inputs that either match expectations that can satisfy set goals or unexpected and salient input that forcefully break through consciousness because of their potential threat or reward. Only a small fraction of the original input through the senses survives the combined attentional filters of bottom-up largely involuntary and top-down partly voluntarily biases to reach awareness; much of it is quickly forgotten. The range of mechanisms that handles these complex filtering processes is what we call attention. As James originally acknowledged the foundation of volitional action is control of attention and the very stream of consciousness he introduced to describe the process is therefore partially determined by the control of attention (James, 1892). It is only through the attention-driven distillation of inputs and their processing and matching with memory templates that decisions can be taken, which reflect the survival constraints of each situation and the overall needs and goals of the individual. Effective control of perception and action demands that the attention focus and its changes are managed by one entity, a realization that prompted many theorists to postulate that the ownership of the attention movement defines consciousness (Taylor et al., 1999). The relationship between attention and consciousness also defines what is excluded from consciousness, and it has been suggested that it serves as a “stable arena for our actions” without the “confounding influence of self-produced motion of multiple receptor arrays mounted on multijointed and swiveling body parts,” so “consciousness arose as a solution to problems in the logistics of decision making in mobile animals with centralized brains, and has correspondingly ancient roots” (Merker, 2005).

Attention is not a unitary process, it is subserved by a range of neural networks that link frontal and parietal areas. Partially segregated systems have been proposed for goal-directed and stimulus-driven attention (Corbetta and Shulman, 2002). These two systems do not act in isolation but they interact in a flexible way to enable dynamic control of attention to the external world to balance the ongoing processing of stimuli relevant to top-down goals and the bottom-up excitation produced by other salient stimuli (Vossel et al., 2014). Two related large scale systems have been identified and found to work in opposition, the salience network, anchored by rostral anterior cingulate cortex (rACC) and an executive network linking dorsolateral frontal and parietal cortices (Seeley et al., 2007). These and other studies of the connectivity structure of the resting state identified a large number of intrinsic connectivity networks (ICNs). These ICNs emerge naturally from the analysis of resting state fMRI data, and remain robust under different mental states (Damoiseaux et al., 2006) including sleep (Horovitz et al., 2009) and loss of consciousness (Greicius et al., 2008; Vanhaudenhuyse et al., 2010). Menon highlighted the significance of three of the ICNs suggesting that aberrant salience mapping and cognitive dysfunction within these three large scale networks are related to a range of psychopathologies (Menon, 2011). The first two ICNs of this triplet, is the pair of anti-correlated networks mentioned above. The first member of this pair is the Central Executive Network (CEN) dealing with executive functions “such as planning, decision making and control of attention and working memory.” The second member is the much discussed default mode network (DMN) (Raichle et al., 2000; Greicius et al., 2003). The last member, the saliency network, is another large scale network responsible for “detecting and orienting to salient external stimuli and internal events” and hence switching between the anti-correlated pair of CEN and DMN.

The networks described above have already been identified “as potential targets of neurofeedback” (Ros et al., 2013; Hamilton et al., 2016; Sitaram et al., 2016). So far, these discussions have mainly focused on the role of attention at the level of assimilation in the awake state. The top down operations of CEN implement actions that are consistent with the current wishes and drives: CEN operates as long as external constraints defined by the prevailing conditions in the environment are (evaluated as) safe. The CEN is allowed to operate without constraints as long as the SN does not detect any danger with its two main nodes playing a sentinel role with the rACC monitoring the external environment and the anterior insula the internal environment. Studies with EEG augmented the role of rACC showing that its activity also predicts the outcome of actions and sends alarms when outcomes do not agree with expectations or conflict arises in the planned order of tasks (O’connell et al., 2007).

### The Default Mode Network, Sleep Architecture and the Core Self

Before the workings of assimilation and accommodation during sleep can be discussed, some basic properties of sleep and some relevant recent results must be reviewed. Humans spend about a third of their lives sleeping. The normal sleep in a night is divided into 4–6 cycles. Each cycle is separated into two main parts, one characterized by Rapid Eye Movement (REM) and the non-REM part (NREM). Within each cycle, there is a progression of sleep stages beginning with two stages of light sleep (NREM1 and NREM2), to deep sleep [two stages NREM3 and NREM4 in (Rechtschaffen and Kales, 1968) or NREM3 and NREM4 amalgamated into one stage in a newer classification (Silber et al., 2007)] and finally REM sleep stage. Each sleep stage is defined by its very characteristic hallmarks: highly rhythmic and/or high amplitude events that produce the tell-tale EEG signatures of each sleep stage. Throughout NREM sleep, there is a fundamental long-lasting sequence of two patterns of EEG activity that closely relates to fluctuations of arousal suggesting that even within the same sleep stage there are two distinct functional states of arousal control mechanism (Terzano et al., 1985, 2001; Ferri et al., 2006; Wehrle et al., 2007). This “cycling alternating pattern” (CAP) gathers together periods of high excitability, which for NREM2, contain the highly rhythmic spindles and high amplitude K-complexes (KCs) events and the more quiet periods in the EEG record, which we labeled as “core periods” (Ioannides et al., 2009). The core periods are unremarkable: they are low amplitude segments of data with no large EEG events and clearly separated from high voltage graphoelements of each sleep stage (e.g., spindles and KCs for NREM2). The core period’s membership to a sleep stage is defined solely by the nature of the periods that precede and follow: if a core period is sandwiched between two periods that through their characteristic EEG hallmarks belong to the same sleep stage, then the core period between them also belongs to that sleep stage.

In our 2009 study (Ioannides et al., 2009) we contrasted the core periods of each sleep stage to those of each other sleep stage and to quiet periods of the awake state with eyes closed, before sleep onset. Separate statistical comparisons were made for the estimates of brain activity derived from wide band MEG data (3 – 200 Hz) and also for the same data filtered in the gamma band (25–90 Hz). The comparison for the wide band data showed highly consistent and highly significant changes established with sleep onset and during deep sleep and REM. These changes were more organized than the corresponding changes derived from the study of the characteristic graphoelements of each sleep stage, but they still failed to reveal any clear continuity as the sleep cycle progressed. The analysis of the gamma band revealed for the first time the much sought after continuity in a few well-circumscribed areas. It was expressed most prominently in two areas located slightly on the left side of the dorsal midline sagittal cut. For reasons that will become apparent later, these two areas are labeled as the midline self-representation core (MSRC) of the brain. The first area, MSRC1 is on dorsal medial prefrontal cortex DMPFC and the second area, MSRC2, is in the precuneus in the midline posterior parietal cortex. High gamma band activity during REM was also reported in other studies and notably related to dreaming (Voss et al., 2009). In this study subjects were trained to become lucid dreamers and to signal lucidity through a pattern of horizontal eye movements. Using this method the authors were able to demonstrate that lucid dreaming had REM-like power in the δ and θ bands and higher than REM power in the gamma band, with the between-states-difference peaking around 40 Hz. The power in the 40 Hz band was strongest in the frontal and frontolateral region, consistent with activity in MSRC1.

**FIGURE 1:**
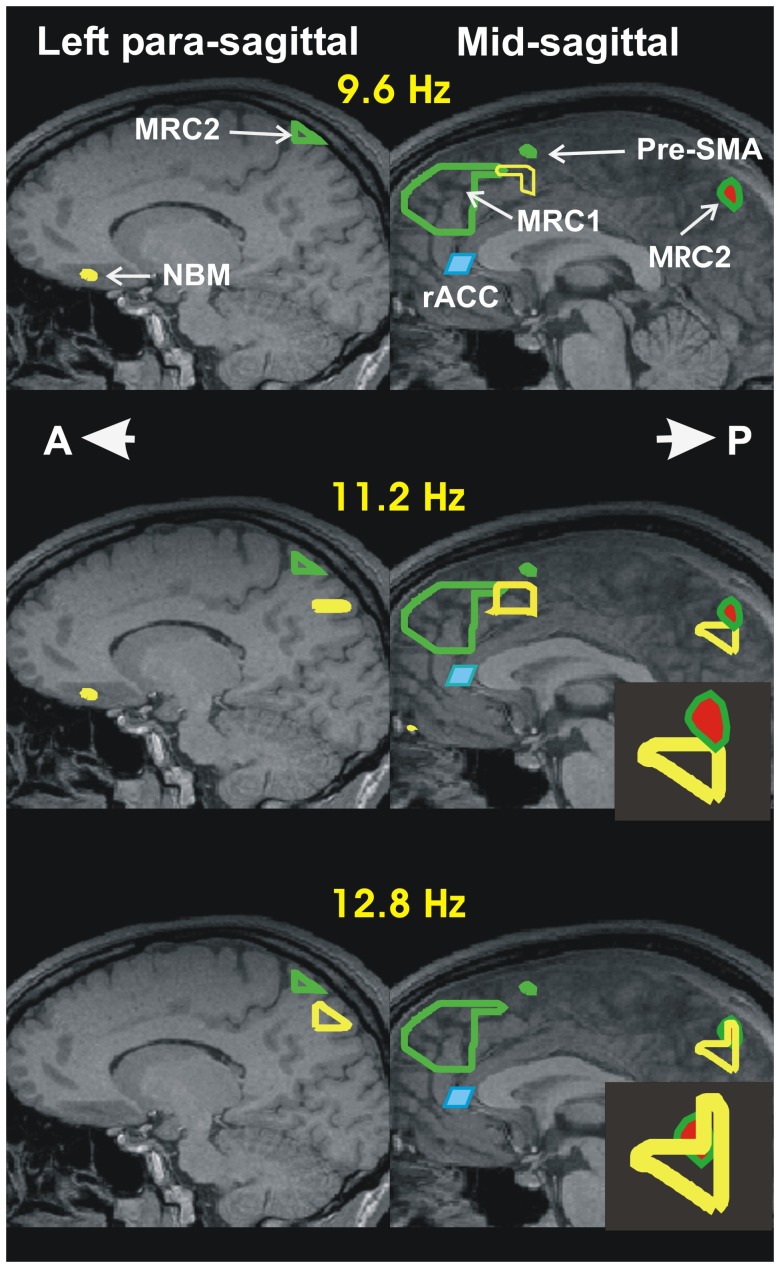
Summary of accommodation related results from our two recent sleep studies: I- Increase in activity during spindles relative to MSRC. The results are displayed in the left paramedial (left column) and mid (right column) sagittal planes. The continuous green outlines are the boundaries of the areas with highest spectral gamma band power, during the quiet periods of REM (Ioannides et al., 2009). It is these two areas that are identified as the two main nodes of the MSRC core of the DMN. The node in the frontal lobe, MSRC1, is captured only in the mid sagittal slice. The node in the posterior parietal cortex, MSRC2, is captured in the paramedial slide and in the mid-sagittal slice (the red filling indicates that it is identified at slightly lower significance). The changes during spindles are presented by the yellow outlines showing the areas of increased activity in the comparison between NREM2 core periods and the 2 s beginning with the start of spindles. The increase in power is confined to the alpha and sigma bands (8–16 Hz). For the Figure the increases are computed independently in three frequency ranges each one 3.2 Hz wide, with center frequencies at 9.6, 11.2, and 12.8 Hz and displayed in the top, middle and lower rows, respectively. Before and during spindles (and KCs) only increases in activity are seen compared to the NREM2 core period. The activations seen in the figure in the alpha and sigma band are the main activations seen during spindles. The figure shows the increases in three main areas: In the frontal areas increases in the alpha and low sigma frequency bands are seen in two areas, one dorsally (just caudal to MSRC1 in the mid-sagittal slice) and the other ventrally in the paramedial slice in the basal forebrain close to the nucleus basalis of Meynert (NBM). The third area shows increase in the high alpha and sigma bands and it is located in the posterior parietal cortex. In the paramedial slice (last two rows on the left column) it is distinct from, and clearly below MSRC2. In the mid-sagittal slice it is just below, and still distinct from, MSRC2 in the high alpha band (second row). In the low and high (not shown in the figure) sigma band an additional activation is seen that overlaps with the MSRC2. These details of the mid-sagittal activations are easier to see in the enlarge icons in the second and third row of the second column. The shaded blue parallelogram directly below MSRC1, just in front of the knee of the corpus callossum, is the rostral anterior cingulate cortex (rACC); in the awake state, this area is responsible for monitoring the environment. The results we reported recently (Ioannides et al., 2017), suggests that rACC plays a similar role during sleep. Specifically, this area is inhibited at the start of light sleep, as part of the general increase in delta in NREM1, but it increases its activity in the NREM2 core period in the alpha and low sigma bands, but actively inhibited (increase in delta) in the two seconds before spindles, but not KCs, for details see panoramic view of these changes in Figure 4 of (Ioannides et al., 2017).

**FIGURE 2:**
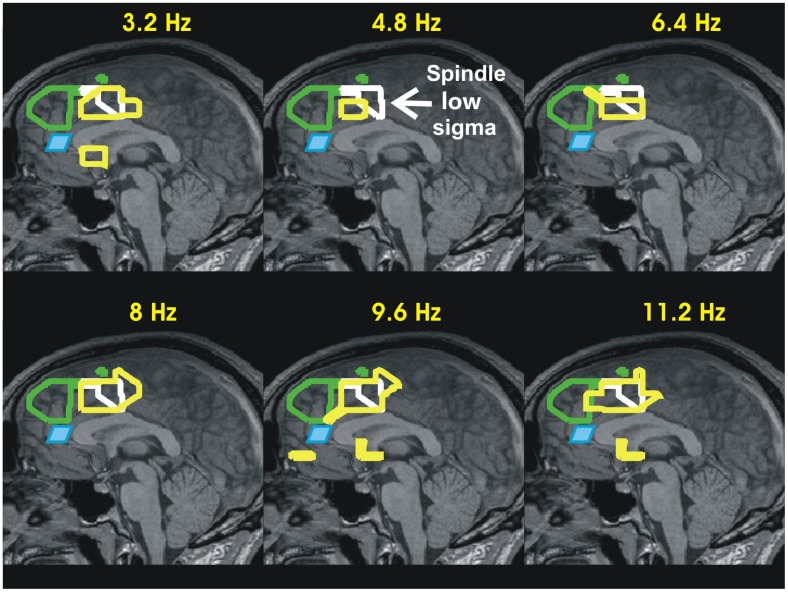
Summary of accommodation related results from our two recent sleep studies: II- Increase in activity during KCs relative to MSRC in the mid-sagittal slice. The results of the earlier sleep study, i.e., the MSRC1 and MSRC2 outlines, and rACC are displayed in exactly the same way as in **Figure 1**. The increases in activity for KCs are displayed as yellow outlines in the mid sagittal plane at six frequency bands of widths 3.2 Hz and centered at 3.2–11.2 with step 1.6 Hz, demonstrating that the increase activity during KCs covers the low frequency ranges too. Additionally a new (continuous white) outline is added, showing as reference the increase in high alpha and low sigma bands during spindles (first and second rows of the right column in **Figure 1**). The results show that in the time leading to KCs the struggle between active inhibition and increase in activity coexists in the area adjacent to that seen during spindles in the frontal lobe in **Figure 1** and just ventral to it, in the cingulated motor area. The KC activity is consistent with alerting influences in the frontal executive and environment monitoring areas receiving both excitatory and inhibitory influences and they are in agreement with proposals for a sentinel role by KCs (Jahnke et al., 2012; Halasz and Bodizs, 2013).

The strength of activity in the gamma band in MSRC1 and MSRC2 increased from awake state to light sleep, increased further in deep sleep and culminated in the highest activity during REM sleep. The MSRC1 and MSRC2 regions, defined by gamma band increases in the core REM periods that are highly statistically significant compared to corresponding activity in the awake state with eyes closed before sleep are shown as green continuous outlines in **Figures 1**, **2**. At a first glance, the location of these two areas, appears to be part of what is now called the DMN. A more careful examination combined with a meta-analysis of results of earlier DMN studies leads to a more interesting conclusion (Ioannides et al., 2009): if the peaks of activity identified in putative DMN tasks are plotted together, they appear to cover wide areas in frontal and parietal areas. However, if only the peaks close to the midline sagittal segment are plotted, they separate into two clusters: one in the frontal cortex and one in the parietal cortex. Each of these clusters is rather dense with an empty region at its center. These two voids are perfectly filled by the MSRC1 in the anterior and by MSRC2 in the posterior DMN cluster. The closest focus to MSRC1, a little over one centimeter away, was identified in conditions reflecting a negative personal experience, contrasting using first person, “I,” with using third person, one’s own name (Moser et al., 2017). In the posterior part, the closest focus to MSRC2 was identified in a combined EEG-PET study with regional cerebral blood flow (rCBF) linearly correlated to the P3 response evoked when subjects were hearing their own first name (Perrin et al., 2005), just a few mm away. In summary, the DMN areas are organized into two main blobs, one in the frontal and the other in parietal cortices. Each of these two blobs separates into areas that appear in tasks involving self-referencing or getting in the mind of others and areas that simply light up when there is no active task. When these results of DMN meta-analysis are combined with the focal gamma band increases identified in the 2009 sleep study (Ioannides et al., 2009), i.e., the MSRC areas, then a three layer onion structure emerges with the MSRC network at its core: with MSRC1 at its anterior and MSRC2 at its posterior pole. The outer most layer is best activated with tasks of maintenance of the state when no imminent action is needed, the classic DMN proper resting state conditions (Greicius et al., 2003). Increased activity in areas of this layer is associated with tasks that do not involve analysis of input from either the senses or introspection. Increase activity in areas of the intermediate layer, closer to the core, are associated to accessing self-referential information, meta-cognition and autobiographical memories; this system deals with tasks usually referred as Theory of Mind (ToM) tasks (Gallagher and Frith, 2003). The core itself shows best during sleep but it begins to show in tasks where fine separation between first and third person descriptions are needed (Perrin et al., 2005; Moser et al., 2017).

The idea for a dedicated subset of the DMN, with nodes close to the mid-sagittal plane devoted to operations related to the self has been proposed many times before. Different authors emphasized the processing of self-referential stimuli (Kelley et al., 2002; Northoff and Bermpohl, 2004), the evaluation within a social context, the representations of self and others (Uddin et al., 2007), and very recently in a meta-analysis of DMN studies (Davey et al., 2016), to name a few. In these studies, the experiments usually use resting state fMRI with tasks where action related to self are used, e.g., remembering past actions and episodes, comparing with others, identifying its facial features etc. The areas identified invariably lie in a layer outside the core of the onion pattern, beyond the core network anchored on the two key areas, MSRC1 and MSRC2. We proposed that this, the MSRC network, is the closest we are likely to come to a neural representation of the core-self, and this is why we named it the midline self-representation core.

### Attention, Memory and Reconstitution of Self During Sleep-Mediated Accommodation

The scene is now ready to resume our study of the assimilation/accommodation process during sleep. We have seen that in the awake state assimilation has the lion’s share of action. The evidence to be presented next shows that during sleep, the rudiment of assimilation is still operating, but this time as a sentinel for danger. Only when all is safe processes related to accommodation can take center stage.

The tomographic analysis focusing on the changes encountered in the core states of light sleep, with emphasis on the emergence of spindles and KCs has been completed recently (Ioannides et al., 2017). **Figures 1**, **2**, summarize the key results relating the activity during spindles and KCs in NREM2 (Ioannides et al., 2017) together with the results from the earlier sleep study (Ioannides et al., 2009) that we used to define MSRC1 and MSRC2. The new analysis for light sleep demonstrated that at the transition from awake state to NREM1, as consciousness is dissolved, two distinct and widespread changes take place: in the frontal lobe regional changes are marked by active inhibition of large areas (seen as strong increases in delta and theta band activity), suggesting reduced executive control and diminished monitoring of the environment; in posterior brain areas regional changes are marked by activity reduction in the alpha and sigma band power in ventral posterior, occipitoparietal and sub-cortical areas (mainly dorsal brainstem). In NREM2 these changes become more widespread, but high frequency activity returns in some frontal areas that specialize in environmental monitoring, notably the rACC. These alerting mechanism leads to KCs as a final check if some danger is detected or to a suppression of all external inputs that allows the spindles to emerge, which many have associated with a role in memory consolidation (Diekelmann and Born, 2010). As can be seen in **Figure 1**, during this process, the key areas activated in the alpha and sigma range of frequencies are adjacent to the MSRC1 and MSRC2, and in the basal forebrain. In the framework we propose, the activation of the key MSRC nodes and the adjacent areas is part of the accommodation process that allows some modification of the neural representation of self. The basal forebrain activation adds a new twist. In the awake state, activations involving the basal forebrain are associated with sharpening sensory input (Everitt and Robbins, 1997; Goard and Dan, 2009; Pinto et al., 2013); since during spindles external input is blockaded, the basal forebrain activation must involve sharpening of internally generated representations, presumably of the new memories to be consolidated.

**FIGURE 3:**
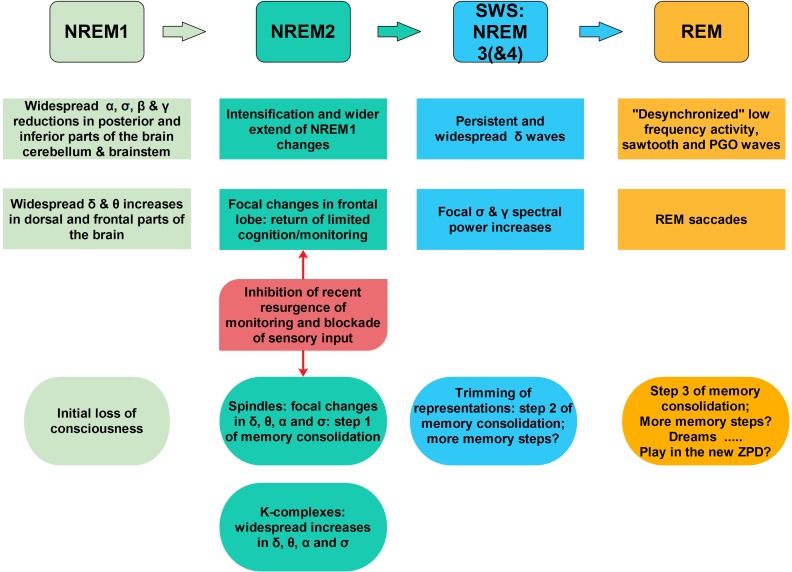
A sketch of the sequence of key accommodation-related events during sleep. As one moves from awake state into light sleep (NREM1) the frontal lobe is actively inhibited (increase in delta band spectral power) while the posterior areas the spectral power is reduced in the alpha and higher frequency bands, entirely consistent with signal changes along the anterior posterior midline in the EEG (De Gennaro et al., 2001) and MEG (Ioannides et al., 2009, 2017). This general trend continues and intensifies during NREM2, except for focal increases in the alpha and low sigma bands in a few foci in the frontal pole, and specifically in the rACC (Ioannides et al., 2017). These changes reveal an element of environmental monitoring has returned amidst the general loss of executive function. From this point on a bifurcation takes place, with one scenario pushing toward spindles and the other toward KCs. In both scenarios, i.e., for both spindles and KCs, only increases in activity are encountered. In the comparison between the 2 s period before spindles with the core period of NREM2 only focal increases low frequency spectral power are encountered (mainly in the delta band) in the frontal lobe. The rACC is one of the few areas inhibited in this way before spindles. During spindles focal increases in spectral power are identified, in the alpha and low sigma bands in the areas adjacent to the MSRC main nodes and in orbital frontal cortex (OFC, BA11) and basal forebrain, in the anterior region of nucleus basalis of Meynert (NBM). Before and during KCs widespread increases are encountered in the delta, theta, and alpha band in the frontal lobes. These increases suggesting a struggle between inhibition and excitation in the frontal lobes. In the 2 s before KCs there is no inhibition of rACC (no spectral power increase in the delta band), as it was seen in the pre-spindle period, suggesting that the return of environmental monitoring seen in the NREM2 core period is maintained. Significantly, during KCs increases are identified in large areas of frontal lobes, including rACC in the delta band and in higher frequencies, consistent with intensification of the struggle between inhibition and excitation of frontal lobe executive function. For spindles, the chain of events seems to lead to a main process that requires cooperative activity between the ventral structures and basal forebrain, as well as activity in the sigma band which links the DMPFC (MSRC1) and the posterior parietal areas (MSRC2). The process of accommodation continues in deep sleep with trimming of connections and further processing during REM tonic phase (Andrillon et al., 2017) and possibly the running of likely extreme scenarios in the newly defined ZPD, making dreams a kind of play preparing for the following day’s struggles.

Reviewing the entire process, as described in **Figure 3**, it becomes evident that many precautions are taken before spindles proceed, from the onset of sleep and through light sleep culminating just before spindles, with the inhibition of MSRC1 and the rACC, the key node of the saliency network that controls DMN and hence MSRC1. Only when these precautions are completed, and all external input is blocked, the process is allowed to proceed. This would make sense if during spindle activity the memory consolidation process is susceptible to external input, i.e., the brain can learn external input during sleep. There was much speculation for this, but only recently clear evidence was provided (Andrillon et al., 2017). It was demonstrated that exposure to samples of novel acoustic noise presented to sleeping human listeners can be “recalled” when the subjects woke up next morning. The improvements in behavioral performance upon awakening (enhancement of learnt acoustic noise sequences and the learning of new ones) takes place during light and REM sleep. Strikingly, the same exposure during deep sleep leads to impaired performance upon awakening. There was a strong and positive correlation between the percentage of trials containing slow frontal spindles and the neurophysiological markers of learning upon awakening, but no correlation when considering fast centro-parietal spindles.

## The Living Self Within and Beyond the Zone of Proximal Development

The ZPD is a key concept introduced through Vygotsky’s work; despite its extensive use it is known in the West through translation of more than one base material that were originally written in Russian (Vygotsky, 1978). In simple terms, the ZPD is defined as the difference between what a learner can do with guidance from a teacher or with more capable peers, beyond what he can do alone (Vygotsky, 1987). We adopt this as a starting point adding a neural underpinning to the terms: ZPD is what the existing neural networks can do but have not done so yet; in principle actions in the ZPD are likely to require no change in the basic networks and specifically no change in the MSRC. A person may not be able to execute a new problem, even if a solution is amenable within his/her current ZPD, if he never encountered this problem before, or if he has encountered it in a different context. A solution can be found with some probing by a teacher or peers, either with hints or even, by simply saying something like “come on you can do this!”. The solution within the ZPD can also be arrived under a forced choice reaction task and during play. The effectiveness of such simple instructions demonstrates vividly why a neural representation of self can endow a social species with a huge evolutionary advantage. A further hint supporting the MSRC with the neural representation of self is the recent report that current stimulation in the lower gamma band during REM induces ongoing brain activity at the stimulation frequency at low gamma frequencies (peak around 40 Hz) that was accompanied by increased self-reflective awareness as measured by the lucid dream index (LuCiD scores) (Voss et al., 2013). We consider next how the new framework with the concept of a neural representation at its center can be used in practice, a topic, however, that we will only briefly touch upon in the remainder of this subsection.

It is possible to provide quantitative description of the ZPD under specific tasks and conditions. For any healthy individual, a measure of his/her normal physiological range of brain activity (n-PRoBA) can be defined objectively from measurements in a series of experiments designed to probe specific brain areas and networks. Such measurements can include behavioral responses (accuracy and reaction time) in specific tasks and/or the electrical activity of the brain in the same tasks or in different resting states while the subject is awake or asleep. Such measurements can be obtained for specific populations so that canonical values for n-PRoBA can be defined for specific age groups, according to gender and condition (e.g., normal subjects and patients with specific pathologies). The behavioral results of a carefully selected set of tests and electrophysiological measurements can be recorded between distinct groups, or within groups after specific interventions allowing the quantitative definition of corresponding changes of n-PRoBA. These changes of n-PRoBA can be used to define the ZPD in an objective and quantitative way. For the standard definition of Vygotsky, the quantitative description of ZPD can be defined as the differences in corresponding n-PRoBA measurements when a child is attempting to solve a series of problems without any help and when guided by a teacher (or helpful suggestions on a screen). Our definition has three advantages. First it provides a quantitative way of exploring the ZPD. Second, it allows the ZPD to be defined across all ages. Third, it extends the range of applications of ZPD into between group comparisons and for following individuals and groups longitudinally. We will describe one such example in the next section.

With the above definitions we can add one more neuroscientific mantle to the other two basic concepts. During routine assimilation, brain activity remains firmly within n-PROBA, with only small excursions into the ZPD. During an accommodation process a controlled excursion is taken away from n-PROBA, well within the ZPD; such an excursion could lead to a re-adjustment of the internal model of the world with some (mild) alterations of our own self-image.

### Preservation and Maintenance of Self Within Its Zone of Proximal Development

A simple principle is proposed that provides a foundational framework for the large scale organization of brain networks both during awake state and sleep: the processes of assimilation and accommodation as described in section “A Theoretical Framework,” are organized so that they ensure the survival and nurturing of the body while maintaining, preserving and allowing slow and controlled changes to the neural representations that help us to face the challenges of life, and especially the MSRC core network that creates the unifying concept of the self. The emergence of the neural representation of the self is then seen as a consequence of evolutionary pressure, a progression from a “prior evolution of a mindreading system which is then directed toward the self” (Carruthers et al., 2012). Increase in the complexity of the interaction with the environment demanded planning beyond simple moment to moment reactions to external events. The internal representation of the environment eventually became detailed enough to include a vague representation of the self. This neural representation of self, once developed endowed the organism with multiple advantages and very importantly it provided a coherent anchor for safe navigation through a dangerous environment (physical and social). In evolutionary terms, it is important that the core self remains intact while its response to the challenges of each day can vary. Retaining the original core (genetic) profile of self is necessary because the variability in the genetic pool must be maintained to allow optimal selection to be exercised as needed in a constantly changing environment; this was indeed the conclusion of early genetic studies (Tryon, 1934) and even earlier observations by Pavlov (1927). Jouvet attempted to explain these observations by suggesting that during REM sleep, such a reprogramming back to the genetic self takes place (Jouvet, 1980). In addition, the consistent behavioral phenotype must have been a tremendous boost to the social network as individuals could be trusted to behave consistently day after day both to each other and when working together in a group. Thus evolutionary selection favored the development and then the sharpening of specialized networks for the representation of self that could maintain themselves (and hence the neural representation of self) in the presence of the vagaries of the environment. These networks could only be viable if they also continued to allow changes when exceptional events that had to be somehow integrated in a very precise and controlled way in the existing internal representations. Looking at the processes of assimilation and accommodation from this point of view we find a nice justification for the network organization that recent studies have revealed. The neural representation of self, carried out by the MSRC core system at the center of what people labeled as the DMN, must be kept dormant during awake state when the environment demands immediate action to avoid danger and grasping of fleeting opportunities. Allowing the self to interfere demands slow introspection, but there is no time for it. Further, the integrity of self will be diluted if its internal representation was continuously eroded by decisions and evaluations of outcomes. Instead, action is delegated to the proxies of self, the areas around the key nodes of the MSRC in the inner rings just beyond the MSRC core. These areas do not represent the self but have access to the wishes and goals of the self as these are shaped by recent history. Thus there is a very good justification for the anti-correlated activity of DMN and the sensory and attention related networks. Until very recently we had only snippets of hints about how accommodation, as defined here, is implemented in the different sleep stages. New studies are needed to help us unambiguously clarify exactly how the accommodation process is completed during deep sleep and REM. Nevertheless, our earlier results (Ioannides et al., 2004, 2009, 2017) and the work of many colleagues coupled with the results of the very recent study of Andrillon et al. (2017), provide enough hints for proposing a plausible scenario for the accommodation process during sleep. This scenario is described in **Figure 3**. In summary, the modification of the neural representation of self, even in a small way, takes place when the environment input is blockaded and in the process the context and meaning rather than the details are retained. If this is true, then the developmental trajectory of dreams will be correlated with the ontogenesis of self, in agreement with Domhoff’s conclusion that “Dreaming is a cognitive achievement that develops gradually over the first 8 or 9 years of life” (Domhoff, 2001). The follow up statement that “dreams are the accidental by-product of two great evolutionary adaptations, sleep and consciousness,” can, however, be modified: dreams are evolutionary adaptations that co-developed with sleep and consciousness. The proposal here is that dreams is the last step in the process of accommodation: during dreaming the self is allowed to play in the REM sleep’s playground with wild excursions in the new ZPD that accommodation has just created in NREM sleep through the incorporation of new memories during NREM2 (Schabus et al., 2004) and trimming of old ones during NREM3 (Andrillon et al., 2017). This play during sleep, just like plays in awake state, are meaningful: they prepare us for likely and demanding tasks of the future, and may be not so likely but potentially critical encounters. Through these plays in sleep, the renewed self prepares and sharpens its servant wishes and goals for the next day’s encounters in an uncertain environment, while the self itself will lie dormant during much of the day.

### Beyond ZPD: Ontogenesis and Disturbances of Self and the Mothering Effect

Because the environment is often harsh and social conditions in adult life often even harsher, staying within the ZPD is not always possible. It is even more difficult, almost impossible to stay within the ZPD, for a newly born baby. When a baby is born it has no complete identity as it has no history of independent existence. A baby is totally dependent on a supportive environment. Support of emotional needs is as important as support of physical needs. The physical separation from the mother, with the cut of the umbilical cord, might constitute the beginning of life as an independent physical entity, but the newborn has yet no identity and no self to rely on. In these early stages there is no assimilation, or if there is some, it is rudimentary. New experiences are hard to integrate since the unifying self is absent. One can consider the somatosensory and motor homunculi starting in the womb (Khazipov et al., 2004), as the primitive progenitor, the scaffolding, on which the self will be assembled. On this scaffolding, the self begins with the sense of agency that controlling body and limbs gives (Delafield-Butt and Trevarthen, 2015). Accommodation and therefore sleep prevails, as would be expected if sleep is essential for the process of accommodation to be successfully implemented. Sleep is also disturbed with nightmares, not an unusual phenomenon as the conflict between the not-yet fully formed self and the day’s events are more likely to be difficult to reconcile. Thus, the process of self-emergence is slow and arduous precisely because the self is not there to begin with. The emergence of self goes hand in hand with control of attention and establishment of memory, as documented by James and Piaget and the developmental psychologists that followed them (Whitebread and Basilio, 2012).

At the very beginning it is the mother’s interaction with the baby that eases the way through a process of shared imitation of each other’s actions. However, this imitation is not passive incorporation of new experiences; it is more a “remodeling and integration of components already in spontaneous expression” (Trevarthen, 1979). The mother senses what is produced and by selecting from the baby’s repertoire for what is to be imitated, she guides the baby to the process of assimilation and accommodation. The mother is more than just observing the babies actions and imitating them; it involves immersion in the joint intention to establish communication and decoding and sharing the emotion of the baby (Lenzi et al., 2009). To achieve this in real time, the mother must analyze amazingly fast every nuisance of body movement, facial expression and intonation of voice; this is a skill so finely tuned by evolution that even exactly how the baby is held in the mother’s arm is biased: the baby’s face is placed in the quadrant of the visual field of the mother where decoding of the baby’s facial expression of emotion is optimal (Liu and Ioannides, 2010). The mothering effect that I just described is then the precise, very fast and continuous feedback the mother gives to the baby, based on empathy and the decoded messages derived from her (sub-conscious) observations of the body posture, facial expression and voice intonation of the baby. In this way, the mother imparts some of her own self to the newly emerging self of the infant. If this elaboration of the theoretical framework of section “A Theoretical Framework” is correct, one would expect major changes in activity to be identified in the general area of MSRC1 and MSRC2 in the perinatal period and the 1st months possibly years of an infant’s life. During the last decade, experiments using resting state fMRI were performed to determine if resting state networks identified in adults are present at birth and even preterm infants; if so, how do these networks change in babies and young children. Most of these infant studies have focused on changes in the DMN. The main findings from some of the key studies are gathered together in **Table 1** and they are summarized by referring to their location relative to the MSRC core nodes identified in our earlier MEG sleep studies (Ioannides et al., 2009, 2017). All infant studies show two key areas either already present in the third trimester or emerging as the first elements of an immature DMN. One area is in the dorso-medial prefrontal cortex, usually referred to as MPFC and the other in the midline posterior parietal, posterior cingulate and restroplenial cortex. The areas identified early on are more anterior and ventral to our MSRC1 in the frontal lobe and more ventral and deep than the MSRC2, they very likely correspond to the outer shells of the DMN. The methods used for the identification of the MSRC core nodes from MEG data (Ioannides et al., 2009) and the fMRI resting state decomposition used in the recent infant studies make it difficult to push the comparison further. Nevertheless, the results from these recent studies are entirely consistent with rapid changes of the DMN that we view as the first steps toward the maturation of the MSRC network. More studies, specifically aiming at disentangling the MSRC core of the DMN are needed. The synthesis of results in **Table 1**, suggests that the DMN and therefore very likely the seeds for the neural representation of self are planted in the womb of the mother. If this is indeed the case, then the evolutionary demand to maintain intact the genetic form of the self, admits an alternative solution to the REM sleep reprogramming (Jouvet, 1980): the core-self together with the body map is established in the womb (Khazipov et al., 2004), and given the absence of any driving input from the environment it is dictated by spindle bursts stamped by the genetic imprint defining its driving early motor activity; this early form of neural representation of self is vague and malleable, as it should be, to fit any one of the many possible environments the infant will encounter at birth. The self is formed and continuously modified early on, especially by the mothering effect in the first few months may be 1st year of life outside the womb. After that it is protected with only minimal changes allowed and only when necessary. These changes are implemented through the accommodation process as defined in sections “A Theoretical Framework” and “The Living Self Within and Beyond the Zone of Proximal Development.” It is perfectly feasible to test these speculations with EEG of sleeping infants.

**Table 1:** The evolution of the DMN in ontogenesis and its relation to the MSRC.

Study (first author and year)	Age of subjects in result(s)	Where area is identified relative to MSRC areas				
		
		Relative to MSRC1		MSRC	Relative to MSRC2	
				
		Ant and/or Ventral	Post and/or ventral		Ant and/or Ventral	Post and/or Ventral
Doria et al., 2010	Preterm	√	X	X	X	X
	Term equiv.	√	√	√ (MRC1)	√	X
	Term control	√	X	X	√	X
Cui et al., 2017	Preterm	√	X	X	X	√
Gao et al., 2009	Term control	√	X	X	X	√
	1 year old	√ (Lateral)	X	X	X	√ (Lateral)
	2 years old	√ (Lateral)	X	X	X	√ (Lateral)
	Adult	√	X	Just anterior to MSRC1	X	√
Ioannides et al., 2009	Adult	See **Figures 1**, **2**		MSRC1	See **Figures 1**, **2**	
				MSRC2		
Fair et al., 2008	Adult	√ (Lateral)	X	X	X	√ (Lateral)
	7–9 years old	X	√ (Lateral)	X	X	X


*During the last decade a series of experiments have used resting state fMRI to determine whether or networks identified in such resting state data in adults are present at birth and even in the preterm and term equivalent age of preterm infants. In some of these studies the networks identified in infant were compared across different ages and/or with networks identified with the same procedures in adults (Fair et al., 2008; Gao et al., 2009). In some studies the range of resting state networks were examined (Fransson et al., 2007; Doria et al., 2010; Cui et al., 2017), while in others the emphasis was placed only on the DMN (Gao et al., 2009). Some studies focused on connectivity analysis using seed areas while other used a more data driven approach using different types of Independent Component analysis. In addition to the uncertainties introduced by these different methods of analysis, the brain morphology in the early years of life changes rapidly and it is distinctively different than that of the adult brain. Nevertheless and despite these differences these pioneering studies provide enough information to allow us to combine the findings in a meaningful way. We first notice that in all these studies the distinct clusters of DMN areas in the frontal lobe and in posterior parietal areas were observed. In the table above, the results are summarized by relating the DMN blobs identified near the midline sagittal brain in the left hemisphere in these studies, relative to the MSRC1 and MSRC2. For each study we mark with a √ if an area is identified and with an X if it is not identified, in a given direction relative each one of the two MSRC key nodes. We add the label “(lateral)” when the identified area in a paramedial rather than medial slide. For the preterm infants, the early evidence initially suggested that at birth there is no DMN (Fransson et al., 2007). For the other studies the details are not entirely consistent, but an overall pattern emerges with the representation of the results in the table above. The earliest activations are seen in ventral regions, mainly anterior-ventral to MSRC1 in the frontal lobe, but also in a posterior ventral direction relative to MSRC2. In general for all ages there is no overlap with MSRC1 and MSRC2, except for one study where the DMN was identified in infants born prematurely but scanned at term equivalent age, which showed the most widely distributed activity.*

During adult life staying within the ZPD is easier than for a baby, provided the passage through infancy and childhood has sufficiently shaped the individual self for the demands of adult life. Key to this is successful social integration initiated through participation in community-wide rituals, music and language (Merker, 2012). Even after a successful maturation of the adult self, dramatic events or persistent pressure from the physical, work and/or social environment can lead to serious deviations away from the ZPD. Isolated excursions can be usually handled over time, but very dramatic events or sustained exposure to physical or emotional pressures beyond the ZPD can lead to all sort of pathological conditions, manifested in a variety of symptoms that often have no apparent common denominator, except disturbances of the representation and maintenance of the image of self; most if not all of these inflictions are accompanied by disturbances of sleep (Morin and Ware, 1996).

## What Neurofeedback Is Like (The Mothering Effect)

In neuroscience, the concept of self is slowly but surely assuming a prominent position with many researchers associating the self with cortical midline structures of the DMN (Decety and Sommerville, 2003; Northoff and Bermpohl, 2004; Uddin et al., 2007; Qin and Northoff, 2011; Wagner et al., 2012). In “A Theoretical Framework,” a more pristine definition of the core self is offered: it is identified with the MSRC, the midline self-representation core. The identification of the neural representation of self with MSRC is based on the observed patterns of brain activity when external input is blocked in very specific parts of sleep, as these are identified from tomographic analysis of MEG data (Ioannides et al., 2009, 2017).

The discussion places the neural representation of self, the MSRC, at the center of the DMN. Within this framework, the common target of NF interventions can be identified as the restoration of aberrant MSRC activity, either directly or through the wider DMN, its anti-correlated CEN or the way these two are managed by the saliency network. The areas around the MSRC are areas having access to properties of the self so that they can report on its history and/or allow templates of action to be released according to the wishes of self (what makes the neural representation of self, consistent with the wider neural representation of the external world).

The framework just introduced suggests that the neural representation of self should emerge in the womb and maturing quickly in the perinatal months, something that is consistent with the results of recent fMRI studies of infants as described in section “Beyond ZPD: Ontogenesis and Disturbances of Self and the Mothering Effect.” The development of self in the early life outside the womb, rely on the intense interchanges of the baby with the mother (Malloch and Trevarthen, 2009). The mother makes the appropriate interchanges, relying on observations (body posture, face expression and voice tone intonation). These observations are the only accessible correlates of the ongoing brain activity, which of course determine the emotional and cognitive state of the baby. We therefore see that under the framework proposed here both NF and the mothering effect address the needs of the self, and this analogy can be pushed a little further with a parallelism of what actually takes place during mothering and NF (using EEG or MEG, NIRS, and fMRI). The mother relies on a complex deciphering of behavioral signs; NF employs direct measurements of selected features of brain activity and provides appropriate feedback to the subject. The mothering effect is directed to a specific baby; NF intervention should be highly personalized. The mother uses the fragments of the rapidly maturing motor actions, components “already in spontaneous expression” (Trevarthen, 1979), to build the first narrative (Delafield-Butt and Trevarthen, 2015); NF uses the existing elements in the observable electrical activity of the brain, directing key features away from pathology toward normality, guided by observations within and between sessions.

What the mother and NF are influencing are large-scale networks in the brain, not individual brain areas or specific frequencies. In NF, modifying the activity of an area or an EEG frequency is not an end in itself; it is only a convenient entry point for modifying the underlying brain networks. In different pathologies, the characteristic modes of brain networks have distinctly different activity levels at key nodes and aberrant power in specific frequency bands. NF often targets the activity of one or more key nodes or frequencies. It seems that often, when the resulting changes are coupled to goal directed belief the entire network can be influenced and directed toward normal levels, with mechanisms relating beliefs and actual brain function that we only now begin to explore (Gu et al., 2016). One may argue whether NF works, but if it does, it is through mild and natural ways of restoring healthy and optimal performance. As such it deserves thorough study, not outright rejection with the label placebo effect. Just like the mothering effect, NF is the first element in a process that involves the rest of the day and especially sleep.

The mothering effect and NF parallelism can guide us on what is or is not reasonable. Imagine an experiment for optimal nurturing of babies with both baby, mother-actor and the experimenters not knowing if the mother-actor and the baby are related. In addition, suppose that the mother-actor is given precise instructions about which types of baby features to use/imitate, e.g., only movements of the right hand to the left. Such experiments are likely to drive crazy both real mothers and babies, and a “cold” analysis of the results would conclude that mothering is not an effective method for nurturing babies! It is precisely for these difficulties that the mother–baby interaction and the way the child develops received so much less scientific attention than what they surely deserve: it is difficult to do meaningful experiments in the lab with strictly traditional procedures. It is for this reason, and key to the theme of this paper, that neither NF nor the mothering effect are ideally suited for the double-blind, placebo controlled studies. This is not an excuse for not performing proper experiments when the resources for doing so are available and ethical considerations allow it (La Vaque and Rossiter, 2001). As already stated in section “A Way Forward From This Conundrum” testing NF with well accepted protocols is necessary, simply because this is the way of getting acceptance and joining the clinical armamentarium (Thibault and Raz, 2016).

What is therefore needed, for both NF and mother-baby interaction experiments, is to identify clearly for what situations the traditional experiments can advance our understanding and for which they cannot. For the later cases new ways of doing scientifically correct experiments must be designed, possibly by adapting existing methods, e.g., similar to the intention-to-treat concept (Gupta, 2011), or developing new ones if necessary. The proposed framework described in this paper can help because it allows a quantitative description of the ZPD based on measurable correlates of neural activity. These can be defined for any age, from infants to old age. For example, the changes in MSRC node activity and connectivity can be studied in infants longitudinally: infant sleep EEG data can be collected before and after an infant – mother interaction and the measurement can be repeated every few weeks or months for the first few years of life.

A similar approach was taken in the Horizon 2020 SmokeFreeBrain project (Horizon 2020 agreement number 681120) that partially supports this work. Two partners in the consortium follow the traditional approach with strict adherence to smoking cessation protocols, one focusing on NF intervention (Greece) and the other on the use of electronic cigarettes (United Kingdom). Our team (Cyprus) uses NF intervention but with a flexible protocol that allows for a wide variation in the details that are selected on the basis of within and between session progress. The development of the framework that I describe here provides the unified platform for relating the results of these three studies through canonical forms that we define through three EEG sets of measurements for each subject. Identical sets of measurements are performed for each subject in the smoking cessation programs of each of the three consortium members and for a set of control subjects. All subjects, including controls have the first EEG measurement. For subjects trying to stop smoking the first EEG takes place at the very beginning before any intervention, and a second and third EEG sessions after 5 and at the end of the full set of (20) NF sessions. The measurements involve resting state EEG and evoked responses to stimuli with some stimuli related to smoking. All subjects complete extensive questionnaires before each EEG experiment. The analysis of the questionnaires and the first set of EEG measurements from control subjects define a canonical set of the n-PRoBA. The analysis of the questionnaires and the first set of EEG measurements from each pool of smokers define the corresponding n-PRoBA for the pool. The difference in the n-PRoBAs of each smoker pool of subjects and that of controls provides a quantitative description of the starting ZPD of the smokers relative to the controls. Repeating the process using the second and third completed questionnaires and EEG measurements provide quantitative measures of (any) changes in ZPD that can then be contrasted with current or future success in smoking cessation. The data analysis will be performed at two stages, first at the level of individual subjects, factoring the details of changes in the protocol taken to optimize the efficacy for each individual and the changes in the beliefs of the subject as these can be extracted from his/her responses to questionnaires, because “beliefs can override the physical presence of a potent neuroactive compound like nicotine” (Gu et al., 2015). This provides a concrete example of the general ideas described in section “The Living Self Within and Beyond the Zone of Proximal Development” for the specific purposes of the SmokeFreeBrain project.

Our analysis converges in its conclusion with recent reviews of NF, notably on its attractive features (Ros et al., 2014): “NFB’s chief strength may not only rest in its direct control of brain oscillations, but in its safety and long-term stability.” The mothering effect and NF are safe treatments because they rely on natural processes. They are both often seen as panaceas because of the generality of their results. In reality they are incomplete interventions: they are the first stage in a process that must be consolidated in the next hours, days and especially during sleep. The term intervention is probably wrong in describing what mothers and NF are doing – they are simply nudging the brain toward its normal physiological condition. The “magic bullet” description is not a suitable for NF; it is appropriate for attempts to restore directly brain activity to normality with no allowance for sleep to act in between or worse still, by attempting to interfere directly with the sleep processes themselves. The possibility, at least in principle, is supported by the recent work of Andrillon et al. (2017), where noise patterns can be learnt when presented during spindles in NREM2 and tonic sleep and unlearnt when presented during slow wave deep sleep. In our view the extraordinary effort expended by evolution to ensure that irrelevant details should not be allowed to interfere with memory consolidation is a warning not to be ignored. Similar concerns can be raised for the effect of nootropic drugs; these drugs do affect the neurochemistry of the brain processes and in doing so they often have a strong effect on sleep. While the use of drugs is necessary in some cases, one might question whether their use as a first line of defense is wise, without monitoring how they impact the basic processes securing that the safety mechanisms evolution has introduced are not compromised. In contrast, NF is linked with the maintenance of the neural representation of self, working synergistically and restoring, when aberrant, the normal physiological processes, recruiting rather than bypassing the safety mechanisms evolution has erected; if this is indeed the case it is surely a methodology that deserves closer attention and if proven effective put as a first rather than last to be tried in the clinical armamentarium, especially when children are involved.

## Notes

This paper is based on the final invited talk with title “Sleep, memory, attention and neurofeedback” at the 6th biennial conference of the Society of Applied Neuroscience SAN2016 that took place in Corfu between 6 and 9 October 2016.

## Author Contributions

AI designed the experiments and carried out and/or directed the analysis of the data from the MEG sleep studies performed at the MEG laboratory he set up and headed from 1998 to 2009 at the Brain Science Institute, RIKEN, in Tokyo, Japan. He worked on the interpretation of the results, synthesis of the studies as described in this paper and drafted the manuscript, and is solely accountable for all aspects of the work.

## Conflict of Interest Statement

The author has set up and works for AAI Scientific Cultural Services Limited (AAISCS), a private research organization focusing on basic and applied research in neuroscience and the foundation of neuroimaging. AAISCS also offers services for signal analysis and neurofeedback. The overall mission of AAISCS is to translate the results of basic research, usually obtained with expensive instrumentation, to clinical applications using simpler instrumentation available to the wider public. The current study is not related to any of the commercial activities of AAISCS. It is part of the basic research program of AAISCS, made possible through funding from Cyprus and European grants, under the overall goal to improve the understanding of promising new technologies that have the potential of improving the health of the wider population at affordable cost.
